# The disruption of the ventricular (VZ) and subventricular (SVZ) zones of the ganglionic eminences in hydrocephalic hyh mice is associated to abnormalities in the cortical GABAergic neurons

**DOI:** 10.1186/1743-8454-7-S1-S57

**Published:** 2010-12-15

**Authors:** Karin Vio, Ana Ojeda, Roberto Yulis, Esteban Rodríguez

**Affiliations:** 1 Instituto de Anatomía, Histología y Patología, Facultad de Medicina, Universidad Austral de Chile, Valdivia, Chile

## Background

The structure of the cerebral cortex results from the orderly migration of two major types of neurons, the glutamatergic projection neurons and the GABAergic interneurons. Most GABAergic neurons originate from the ganglionic eminences. They first migrate tangentially toward the pallium and then migrate radially through the developing brain cortex. The disruption of the VZ and SVZ of the palium and subpalium has been shown to occur at key developmental periods in the hydrocephalic mutant mouse hyh. The aim of the present investigation was to study whether such a disruption of the VZ and SVZ results in abnormalities of the GABAergic neurons populating the brain cortex.

## Materials and methods

The brain of non-hydrocephalic and hydrocephalic hyh mice at postnatal day 7 (n=20) were processed for immunocytochemistry and immunofluorescence using antibodies against GABA and the marker of neuronal nuclei NeuN. Sections processed for double immunofluorescence were inspected with an epifluorescence microscope provided with the multidimensional acquisition software AxioVision Rel. Single and overlay images were used for quantitative analyses of the whole populations of cortical neurons (NeuE-reactive) and that of GABAergic neurons. Absolute and relative cell density and intracortical distribution were recorded for the GABAergic neurons.

## Results and conclusions

The mutant hyh mice were characterized by (i) a marked reduction in the width of the cerebral cortex; (ii) a reduction in the total number of GABAergic neurons; (iii) a reduction in the relative number of GABAergic neurons with respect to the total population of neurons (GABA/NeuE); (iv) an abnormal distribution of GABAergic neurons in the cortex layers, with a significant reduction in layers II and III and an increase in layer IV y V. The hyh mutation is associated with: (i) a decreased number of GABAergic neurons migrating from the ganglionic eminences; (ii) abnormal migration of GABAergic neurons through the developing brain cortex; (iii) in hyh mice disruption of the VZ and SVZ is associated to the onset of hydrocephalus and abnormal corticogenesis.

